# Polyphenol-enriched fraction of *Acacia seyal* Delile (Mimosaceae) inhibits human triple-negative breast cancer cells and exhibits anticancer and immunomodulatory effects in a DMBA-induced breast cancer model

**DOI:** 10.3389/fphar.2026.1772878

**Published:** 2026-06-17

**Authors:** Alain Brice Tueche, Michael Hermann Kengne Kamdem, Jordan Lembe Tonga, Edwin Mpoh Mmutlane, Derek Tantoh Ndinteh, Stéphane Zingue, Dieudonné Njamen

**Affiliations:** 1 Department of Animal Biology and Physiology, Faculty of Science, University of Yaoundé 1, Yaoundé, Cameroon; 2 Drug Discovery and Smart Molecules Research Laboratory, Department of Chemical Sciences, University of Johannesburg, Johannesburg, South Africa; 3 Centre for Natural Product Research (CNPR), Department of Chemical Sciences, University of Johannesburg, Johannesburg, South Africa; 4 Research Centre for Synthesis and Catalysis, Department of Chemical Sciences, University of Johannesburg-Kingsway Campus, Johannesburg, South Africa; 5 Department of Pharmacotoxicology and Pharmacokinetics, Faculty of Medicine and Biomedical Sciences, University of Yaoundé 1, Yaoundé, Cameroon

**Keywords:** anti-oxidant, DMBA carcinogen, IFN-γ cytokine, mammary tumor, metastasis, monocytes, polyphenolic extract

## Abstract

**Introduction:**

Breast cancer killed 665,684 patients globally in 2022 despite government efforts and conventional therapies. The hydro-ethanolic extract of Acacia seyal, a sub-Saharan African ethnomedicinal plant, showed moderate cytotoxicity, significant anti-migration and in vivo preventive effects against breast cancer cells. The aim of this study was to propose an improved traditional extract and to evaluate its efficacy and potential mechanism of action.

**Methods:**

To achieve this, A. seyal hydro-ethanolic extract (ASHE) was fractionated in polyphenol-enriched (ASpo), polysaccharides (ASsu) and residue (ASre) fractions. Fractions along with the crude extract were tested for their ability to inhibit cell growth (MTT) and proliferation (CCK-8). The promising fraction (ASpo) was further investigated on clone formation, caspase-3, wound healing, chemotaxis and cell adhesion to understand its underlying mechanisms of action. Moreover, ASpo at 18.75 and 37.5 mg/kg, *p.o* compared to standard drugs (tamoxifen and letrozole) and control (distilled water) were assessed in a 20-week preventive study of DMBA (50 mg/kg, *s.c*)-induced breast cancer by considering tumor incidence, tumor volume, organ mass, histopathological, hematological, antioxidant and anti-inflammatory/immunomodulatory markers.

**Results:**

Among the 3 fractions, ASpo compared to control, inhibited (*p* < 0.01) MDA-MB 231 cells growth, proliferation and clone formation at 50 μg/mL. It increased caspase-3 and inhibited cell migration and invasion (*p* < 0.01) with increase (*p* < 0.01) adherence to collagen and fibronectin. *In vivo*, comparable to reference drugs, ASpo reduced tumor incidence (50%), and tumor volume from 7827.30 mm3 (adenocarcinoma SBRIII, 10% lymphocytes infiltration) in DMBA to 804.32 mm3 (adenocarcinoma SBRII, 30% lymphocytes infiltration) at 37.5 mg/kg. It increased femur and thymus’mass, lymphocytes and monocyte levels (*p* < 0.001) in serum, reduced (*p* < 0.001) TNF-α, IL-6, IL-12, EGF, nitrites levels whereas increased SOD, catalase and major IFN-γ cytokine (*p* < 0.001). ASpo additionally showed a safety profile on toxicity organs.

**Conclusion:**

Overall, ASpo mainly contributed to anti-breast cancer activities of total crude extract and involved immunomodulatory effects to consider with optimized bioactivity to further investigate it as a promising natural and inexpensive alternative to costly and efficient current immunotherapy.

## 1 Introduction

Breast cancer (BC) is a very heterogeneous malignant disease with three main subtypes encompassing estrogen and/or progesterone receptors positive (ER+/PR + or hormone-dependent) accounting for 65%–70%, human epidermal growth factor receptor two positive (HER2+) representing 15%–20% and triple-negative breast cancer (TNBC) with absence of ER, PR and HER2 amplification (10%–20%) (Benesova et al., 2024). It is the most recurrent cancer in women (11.6% of all cancers) with 2,308,897 new cases and 665,684 deaths globally in 2022 (Bray et al., 2024). BC-related deaths are recognized to be mainly caused by metastasis derived from invasion and migration of primary tumor cells and inducing the lesions to distant organs such as bone, lung, liver and brain (Kuett et al., 2025). The initiation and development of BC involves chronic inflammation linked to oxidative stress with reactive oxygen species (ROS) and inflammatory cytokines such as tumor necrosis factor-alpha (TNF-α), chemokine fractalkine, Epidermal Growth Factor (EGF), interleukine-6 (IL-6) and interferon-gamma (IFN-γ) in the tumor microenvironment (Balkwill and Mantovani, 2001; Dong et al., 2025). TNBC is frequently associated with a high prevalence to BRCA1/2 germline mutations and is the most aggressive BC subtype with higher metastatic potential, limited treatment options and worse prognosis (Blaszczak et al., 2025).

Modern immunotherapy with promising efficacy that provides new hope for BC patients is limited by accessibility and financial constrains ($70.000 or FCFA 42 million for 1 year of reference pembrolizumab treatment), while future directions involve personalized approaches and combined therapies with pembrolizumab (Heater et al., 2024; Zhang et al., 2025). In addition, traditional standard chemotherapy accessible for patients has variable efficacy, serious long-term adverse effects (cardiotoxicity, cognitive impairment, fatigue) and several patients ultimately develop drug resistance (Obidiro et al., 2023). Anti-estrogen medications like letrozole (aromatase inhibitor) and tamoxifen (selective estrogen receptor modulator) are the first therapeutic approach of choice following surgery for the management of patients with estrogen-dependent (ER+) BC in spite of hot flashes as side effects and endocrine resistance from long-term usage (Navarro et al., 2025).

As a result of the side effects and high cost of conventional therapies, many patients with long-term BC and in distress or newly diagnosed because of counsels from relatives or friends, choose Complementary and Alternative Medicine (CAM) (Stöcker et al., 2023). Most CAM which includes natural products lack pharmacological toxicity with documented human intake (Stöcker et al., 2023). Natural products with phytoconstituents or secondary plant metabolites have been increasingly considered as therapeutic and preventive alternatives for estrogen-dependent and triple-negative breast cancers (Bai et al., 2024). Polysaccharides or non-toxic biological macromolecules are other phytoconstituents reported to have anti-cancer activities through immunomodulatory effects (Wang et al., 2023). Polyphenols included in the family of phenols are an important class of secondary plant metabolites that have been receiving considerable research attention as a result of their antioxidant and anti-tumor potential (Moar et al., 2024). Some authors reported substantial anti-tumor effects with polyphenol-enriched extracts from European blueberry, Chinese Tea, American pecan nutshells and Korean *Nelumbo nucifera* leaves in MCF-7 and MDA-MB-231 cells as well as in BALB/c mouse model (Li et al., 2016; Vuong et al., 2016; Wu et al., 2017; Ribas et al., 2023).

In this study we focused on *Acacia seyal* Delile (Mimosaceae) stem barks traditionally used in sub-Saharan Africa for the treatment of various inflammatory conditions including chest pain (pneumonia), joint pain (rheumatic arthritis), skin necrosis nodules (dermatosis) and sarcoma (a type of cancer related to the breast) following Arbonnier (2009) and Ashour et al. (2022). Eltayeb et al. (2017) reported Acacia seyal Delile stem barks contain reducing sugars, steroids, terpenoids, alcaloids and polyphenols (flavonoids, tannins) with ant-inflammatory activities (Sun et al., 2024). Total phenols (90 ± 1 mg gallic acid) including polyphenols and subclasses namely flavonoids (47 ± 14 mg eq quercetin) and flavonols (16 ± 6 mg eq quercetin) were quantifed from *Acacia seyal* hydro-ethanolic crude extract in the previous study (Zingue et al., 2018b). Among polyphenolic compounds of *Acacia seyal* Delile, gallic acid, caffeic acid and derivatives, catechin and epicatechin were reported with anti-inflammatory, anti-oxidant, immunomodulatory and anti-breast cancer activities (Hussain et al., 2016; Khan et al., 2016; Rezaei-Seresht et al., 2019; Elmi et al., 2020; Pereyra-Vergara et al., 2020; Ashour et al., 2022; Andrade et al., 2024; Xie et al., 2024). The water extract of *Acacia seyal* Delile barks showed a moderate cytotoxicity on human fetal lung fibroblast MRC-5 cells at 256 μg/mL (Elmi et al., 2020). The hydro-ethanolic extract from its barks also showed a moderate cytotoxicity on estrogen-dependent MCF-7 (CC50 = 250 μg/mL) and triple negative MDA-MB-231 breast cancer cells (CC50 = 100 μg/mL) by triggering intrinsic apoptosis with an anti-metastatic potential through inhibition of cell invasion and migration (Zingue et al., 2018a). *In vivo*, this extract showed preventive effects against DMBA-induced breast cancer in Wistar rats at the doses of 150 and 300 mg/kg (Zingue et al., 2018b). In the present study, this extract was fractionated in polyphenols, polysaccharides and residue fractions and their anti-breast cancer activities were investigated to see whether this process could be used to optimize its activity. More specifically, *in vitro* these fractions were first screened in cell viability assays (MTT and CCK-8) on ER+ (MCF-7) and TNBC (MDA-MB-231) cells, followed by evaluation of the mode of action of the promising fraction on clonogenic growth, caspase-3 activity and metastatic hallmarks (chemotaxis, migration and adhesion). *In vivo*, the promising fraction was evaluated in a chemopreventive study against DMBA-induced breast cancer in Wistar rats through survival rate, tumor incidence and burden, tumor mass and volume, tumor histoarchitecture, serum levels of inflammatory cytokines (TNF-α, fractalkine, EGF, IL-6, and IFN-γ), mammary stress oxidative makers (SOD, CAT, NO and MDA).

## 2 Materials and methods

### 2.1 Chemicals and reagents

Streptomycin, penicillin, FBS (Fetal bovine serum) and glutamax were supplied by Gibco (Karlsruhe, Germany). MTT dye was supplied by Roche Diagnostics (Penzberg, Germany) while CCK-8 and caspase 3 kits were supplied by GlpBio (California, United States). 6-well transwell chambers were supplied by Greiner (Frickenhausen, Germany). Pre-carcinogen 7,12- dimethylbenz(a)anthracene (DMBA) with at least 98% of purity was supplied by Sigma-Aldrich (Steinheim, Germany). Letrozole from Novartis Access® and tamoxifen citrate from Mylan® was supplied by Salutas Pharma GmbH (Barbelen, Germany). Anesthetics Valium® (Diazepam) and Ketamine hydrochloride® were supplied respectively by Roche Pharma (Fontenay-sousbois, France) and Rotex medica (Tritau, Germany). MILIPLEX® cytokine kit for the technology luminex Xmap® was supplied by Milipore Sigma (Minneapolis, United States). Creatinine and Alanine transaminase reagent kits were supplied by Fortress Diagnostics Limited® (Muckamore, United Kingdom).

### 2.2 Plant collection and preparation of extract and fractions

#### 2.2.1 Plant collection

The sample of *A. seyal* (Mimosaceae) used in this study was collected in Moutourwa (Far-North Region, Cameroon) in March 2015 at the geographical coordinates of 10°22′270″ North, 014°19′686″East and 463 m of altitude with a GPS or Global Positioning System (Garmin, Kansas, United States). The plant was authenticated at the Cameroon National Herbarium (CNH) in comparison with the specimen deposited under the voucher specimen N° 19223 SFR/Cam.

#### 2.2.2 Preparation of crude extract

As described by Zingue et al. (2018a), 2000 g of well-dried and pulverized stem barks of *A. seyal* were macerated in 6 liters of distilled water/ethanol mixture (v/v: 30/70) for 48 h at room temperature. Then 353 g (17.7%) of a brown crude extract was obtained following filtration through a Whatman no 4 paper (Belle France, Dijon, France), evaporation of the ethanol using a rotary evaporator (Heildoph, Schwabach, Germany) under reduced pressure (175 mbar) and lyophilization.

#### 2.2.3 Preparation of enriched fraction in polyphenols and polysaccharides

The polyphenol-enriched fraction of *A. seyal* (ASpo) was prepared using the resin enrichment method described by Zingue et al. (2016). Briefly, 225 g of *A. seyal* hydro-ethanolic crude extract (ASHE) was dissolved in 4 L of distilled water and mixed for 1 h in a water bath at 40 °C with 900 g of XAD-7 PH resin (Sigma-Aldrich, Missouri, United States) previously activated overnight with 95°ethanol. The mixture was then introduced into a separating funnel (2,000 mL) followed by removal of the supernatant and repeated rinsing of the resin with 6 L of distilled water to collect water-soluble fraction of crude extract. The water-insoluble fraction was recovered by passing of 95° (12 L) ethanol repeatedly through the rinsed resin to break the weak Van der Waals and hydrogen bonds between the resin and the aromatic or carbonyl rings of the polyphenols. The water-insoluble fraction collected (polyphenol-enriched solution) was concentrated in a rotary evaporator (Heildoph, Schwabach, Germany) at 60 °C and 175 mbar pressure to obtain 65 g of ASpo with a yield of 28.88%.

Furthermore, the method described by Hua et al. (2014) was used to obtain the polysaccharides fraction (ASsu). Following ASpo, the water-soluble fraction was then dried at 60 °C in a ventilated oven to give 96 g fraction. This fraction was dissolved in 1 L of distilled water and decocted for 3 h in a water bath at 80 °C. The decoction was filtered with n°4 coffee filter paper (Belle France, Dijon, France) and the filtrate obtained was mixed with 4 L of 95° ethanol for precipitation in the refrigerator at 4 °C for 24 h. Thereafter, the 95°ethanol was concentrated at 175 mbar, 60 °C in (Heildoph, Schwabach, Germany) to 5^th^ of the initial volume, followed by centrifugation at 3500 rpm for 15 min using a centrifuge (Hettich, Tuttligen, Germany) in 50 mL tubes (Eppendorf, Hamburg, Germany). The supernatant was collected and the pellet was introduced into a tube and dried in a Vent-linc ventilated oven (Southern Labware, Georgia, United States) at 60 °C. This process yielded 93 g of ASsu from the pellet and 3 g of residual fraction (ASres) from the supernatant.

#### 2.2.4 UHPLC-HR-LC-MS analysis of A. seyal Delile extract and fraction

The fingerprints of the ASpo and ASHE were performed using a Dionex UHPLC (Thermo Fisher Scientific, California, United States) equipped with a Fortis C18 column (3.0 × 100 mm, 3 µm; Fortis Technologies Ltd, Cheshire, UK) at 25 °C with a flow rate of 0.2 mL/min. A guard column (5 mm × 2.1 mm, 3 µm) with the same stationary phase was placed before the column. The mobile phase consisted of mixture of water (solvent A) and acetonitrile (MeCN, solvent B), both adjusted with 0.1% formic acid (FA). The multistep gradient mode was operated as follow: isocratic 10% B for 5 min, 10%–20% B for 5 min, 20%–30% B for 5 min, 30%–40% B for 5 min, 40%–100% B for 18 min, isocratic step for 6 min at 100% B, 100% to 10% B for 0.1 min and a final isocratic step for 6 min at 10% B. The auto-sampler manager was thermostated at 40 °C and the injection volume was set at 5 µL. The HR-LC-MS/MS data were acquired with a mass range of 50–2000 *m/z*. For the HR-LC-MS/MS acquisitions, analyses were performed on a Bruker Compact QToF mass spectrometer using an electrospray ionization (ESI) probe (Bruker, Bremen, Germany). ESI conditions operated in positive mode were as follow: End plate offset = 500 V; Nebulizer = 1.8 Bar; Dry gas = 9.0 L/min; Dry temperature = 220 °C; Capillary voltage = 4.5 KV. Nitrogen was used as nebulizer and dry gas. A method including the detection (full scan) and fragmentation of the most intense peaks per scan was used. Collision energy was varied from 7 to 40 eV. Major secondary metabolites in *A. seyal* Delile extract and fraction were identified using Chromeleon 7 Chromatography Data System software (Thermo Fisher Scientific, Sunnyvale, United States).

### 2.3 Cell bioassays

#### 2.3.1 Cell lines

MDA-MB-231 and MCF-7 cell lines were used for *in vitro* assays. MDA-MB-231 cell line isolated at MD Anderson Cancer Center is a triple-negative epithelial human breast cancer cell line. MCF-7 cell line from Michigan Cancer Foundation is a human adenocarcinoma cell line with estrogen and progesterone receptors. The cell lines were provided by American Type Culture Collection (ATCC)/Laboratory of the Government Chemist (LGC) Promochem (Wesel, Germany).

#### 2.3.2 Cell culture

The culture of MDA-MB-231 and MCF-7 cells was carried out in Roswell Park Memorial Institute 1640 (RPMI-1640) medium with a supplement of 10% FBS, 1% penicillin 100 U/mL and streptomycin 100 μg/mL. The cells were stored in 5% humidified CO_2_, 37 °C and pH 7.4 incubator. The culture medium was replaced by a fresh medium every 2 days and the counting of living cells was performed using the trypan blue reagent and a counter which automatically gave the values.

### 2.4 Evaluation of cell growth/proliferation

#### 2.4.1 Cell growth with MTT assay

The growth of MCF-7 and MDA-MB-231 cells in presence of ASpo, ASsu and ASres fractions was evaluated using mitochondrial tetrazolium test (MTT). These cells in a volume of 100 μL RPMI-1640 and a density of 1 × 10^4^ cells/mL were seeded in 96-well culture plates. A range of concentrations from 12.5 to 200 μg/mL of dissolved fractions in vehicle 0.01% DMSO (dimethylsulfoxide) were added to corresponding wells while control cells received the vehicle only. Following 24, 48 and 72 h of treatment, 10 µL of MTT solution (0.5 mg/mL) was added to each well and incubated for 4 h. Thereafter, 10% SDS (sodium dodecyl sulphate) with 0.01 M HCl buffer were used to solubilize formazan crystals. Lastly, after an overnight incubation in 5% humidified CO_2_ and 37 °C incubator (Thermo Fisher Scientific, Massachusetts, United States), the reading of the absorbance of each well was done at 570 nm using a reader ELISA (Tecan Crailsheim, Germany).

#### 2.4.2 Cell proliferation with CCK-8 assay

The assessment of the effect of ASpo (retained fraction) on cell proliferation was performed using Cell Counting Kit-8 (CCK-8). Briefly, 96-well plates were seeded with 100 μL of mammary cancer cells (MCF-7 and MDA-MB 231 at 1 × 10^4^ cells/mL) and incubated in a 5% CO_2_ incubator for 24 h to attach. Then, 50 and 100 μg/mL of newly prepared enriched extracts in 10 μL of RPMI-1640 was added to different wells while control cells received 0.01% DMSO as vehicle. Following an incubation of 48 h, each well was exposed to 10 µL of CCK-8 solution and allowed to incubate for a further 48 h. Lastly, the plates were homogenized and a microplate reader (Tecan Crailsheim, Germany) was used for the reading of absorbance at 450 nm. The inhibition of cell proliferation has been calculated using the formula: Inhibition rate (%) = [(Ac-As)/(Ac-Ab)] × 100, where: As = absorbance of the experimental well (absorbance of cells, medium, CCK8 and wells of the test compound); Ab = blank well absorbance (absorbance of wells containing medium and CCK8); Ac = control well absorbance (absorbance of wells containing cells, medium and CCK8).

As these cells were the most sensitive, the triple-negative MDA-MB-231 line was selected for subsequent experiments.

#### 2.4.3 Clonogenic assay

To assess the potential of ASpo to inhibit clone formation, 500 MDA-MB-231 cells were seeded per well of 6-well plates. After removal of medium from the plates, the polyphenol-enriched fraction at the concentrations of 25, 50 and 100 μg/mL, was added to appropriate wells and incubated for 7 days. Colonies (50 cells minimum) were counted in treated cells as well as control cells and photographed with a magnification objective of ×40 under a fluorescent microscope Zeiss Axio Observer Z1 (Zeiss microscopy GmbH, Hallbermoos, Germany) following Fotsing et al. (2023).

### 2.5 Caspase-3 activity assessment

Caspase-3 activity was assessed by detection of the chromophore p-nitroaniline (pNA) following cleavage of the DEVD-pNA-labeled substrate (Peterson et al., 2010) using caspase-3/CPP32 (32-kDa putative cysteine protease) colorimetric assay kit. A seeding of 1 × 10^6^ MDA-MB 231 cells/mL in T75 cm^2^ cell culture flask was done and the flask was incubated for 24 to allow for adherence. ASpo was prepared prior to use, added at two effective concentrations (50 and 100 μg/mL) in the different flasks and allowed to incubate for 48 h. Proteins (supernatant) were isolated by centrifugation for 20 min at 4 °C and 12,000 rpm after lysing of cells. Protein concentration was assayed by Bradford method and 100 μL of protein was added to a 96-well plate followed by a dilution using 2× reaction buffer (50 µL) with10 mM DTT. Thereafter, an addition of 4 mM DEVD-pNA substrate at the volume of 5 µL was done for each well with an incubation for 2 h in 5% CO_2_ and 37 °C. Lastly, microplate reader (Tecan Crailsheim, Germany) was used to quantify at 405 nm from pNA light emission.

### 2.6 Evaluation of anti-metastatic potential of Acacia seyal polyphenol-enriched fraction

#### 2.6.1 Cell migration

The potential of ASpo to impede the migration of MDA-MB-231 cells was determined using wound-healing assay. Into 6-well plates, 5 × 10^5^ cells/well were seeded and allowed to attain confluence. Then, the RPMI-1640 medium was substituted with serum-free medium. After 4 h, a 100 µL pipette tip was used to create a scratch wound that induced a mechanical detachment of cells which were eliminated by washing with PBS (phosphate buffered saline) twice. Afterwards, DMSO 0.01% (control solvent) or ASpo at 50 and 100 μg/mL were incubated with cells for 72 h in RPMI-1640 FBS-free medium. An optical microscope (40×) Zeiss Axio Observer Z1 (Zeiss microscopy GmbH, Hallbermoos, Germany) was used to observe the level of recovery of the wound areas by cells in migration. A recording of microphotographs at intervals of 24 h as well as the quantification with ImageJ® software of the wound healing area were done as last steps of wound-healing assay according to Li et al. (2019) and Nguedia et al. (2024).

#### 2.6.2 Cell invasion

The 6-well transwell chambers that have 8 µm pores was used to assess the potential of ASpo to hinder MDA-MB-231 cell invasion (chemotaxis) linked to serum-rich medium. An incubation of MDA-MB 231 cells for 24 h with ASpo at two concentrations (50 and 100 μg/ml) was carried out in T25 cm^2^ flasks. The cells were washed with PBS followed by the addition of trypsin and cell counting. This was followed by placement of 500,000 cells/2 mL in the upper chamber of the insert with a serum-free RPMI-1640 whereas the lower chamber received RPMI-1640 at the volume of 2 mL supplemented with 10% FBS. At the end of 24 h, the non-invading cells on the upper surface of the transwell membranes were delicately taken out with a cotton swab. Lastly, the invading cells on the lower surface underwent fixation with 2% glutaraldehyde, followed by staining with hematoxylin and counting was done using a Zeiss Axio Observer Z1 fluorescent microscope (Zeiss microscopy GmbH, Hallbermoos, Germany) with ×20 magnification in reference to Yi et al. (2020) and Nguedia et al. (2024).

#### 2.6.3 Evaluation of cell adhesion to extracellular matrix

Plastic 6-well plates were coated with collagen or fibronectin (components of extracellular matrix) overnight. In order to avoid nonspecific cell adhesion, the plates were incubated in 1% PBS and BSA (bovine serum albumin) at room temperature for 1 h. Thereafter, MDA-MB-231 cells at subconfluence in T25 cm^2^ flasks were treated for 24 h with ASpo (50 and 100 μg/mL) or DMSO (control solvent). After the treatment of cells, they were washed with PBS followed by the addition of trypsin and counting. This was followed by the addition of 500,000 cells/2 mL into each well for 60 min. To determine the mean of cell adhesion rate, a Zeiss Axio Observer Z1 optical microscope (Zeiss microscopy GmbH, Hallbermoos, Germany) at the magnification of ×20 was used in the final step to count in five distinctive areas the adherent cells fixed in advanced with glutaraldehyde (2%) after washing off of non-adherent cells with PBS as described by Zhang et al. (2009) and Nkuimi et al. (2024).

### 2.7 *In vivo* chemopreventive study and toxicological profile

#### 2.7.1 Experimental animals

The experimental animals were 30–35-day-old female Wistar rats (*Rattus norvegicus*) weighing between 55 and 65 g. The rats were obtained from the Animal Physiology laboratory of the University of Yaounde 1 (Cameroon). The animals were housed in plastic cages at room temperature (about 25 °C) in a natural light and dark cycle with enough ventilation. Tap water and a standard diet without soy were provided to the animals freely and regularly. Following Nkuimi et al. (2024), the animal diet comprised corn 42%, bone meal 3%, wheat flour 22%, fish meal 19%, crushed palm kernel meal 4%, sodium chloride 0.75%, peanuts 9% and multivitamin complex-Olivitazol® 0.5%. Animals were housed and treated as approved by the Joint Institutional Review Board Animal and Human Bioethics of the Faculty of Science (University of Yaoundé 1) in agreement with directives of the European Union on the care of animals (EEC Council 86/609).

#### 2.7.2 Choice of doses and preparations for administration

The pre-carcinogen DMBA for BC induction was dissolved in olive oil according to Yadav and Chumbhale (2022) in a volume of 1 mL and administered at the dose of 50 mg/kg body weight (BW) through subcutaneous route (*s.c*). Tamoxifen (3.3 mg/kg BW, standard drug) and letrozole (1 mg/kg BW, standard drug) were both dissolved in distilled water and given orally at a volume of 10 ml/kg BW (Maltoni et al., 1997; Mvondo et al., 2020). The tested dose of 37.5 mg/kg BW for ASpo was selected based on a preliminary study on the hydro-ethanolic extract of *A. seyal* by Zingue et al. (2018a), and was halved (18.75 mg/kg BW) to assess the dose-dependent effect. ASpo fraction was dissolved in distilled water supported by Elmasonic ultrasonic bath (Elma Schmidbauer GmbH, Singen, Germany) for 10 min (45 °C, 37 kHz frequency) and given orally at the volume of 10 ml/kg BW.

#### 2.7.3 Chemopreventive study design

Forty-eight (48) female Wistar rats aged of 40–45 days after a 10-day acclimatization were randomized into 6 groups of 8 animals each (n = 8). The groups serving as normal control (NOR) and negative control (DMBA) received distilled water. Two other groups serving as positive control received 3.3 mg/kg of tamoxifen (TAMOX + DMBA) and 1 mg/kg of letrozole (LETRO + DMBA), respectively. The ASpo 18.75 + DMBA and ASpo 37.5 + DMBA test groups received the *A. seyal* polyphenol-enriched fraction at a dose of 18.75 mg/kg BW and 37.5 mg/kg, respectively. As reported by Fotsing et al. (2023), all treatments were done by oral gavage daily for 10 days before BC induction and continued until 20 weeks. Apart from the NOR group which received the vehicle (olive oil), all the other groups received a single dose of 50 mg/kg BW of DMBA dissolved in 1 mL of olive oil at the inguinal area. Five hours after breast cancer induction all the groups that received DMBA were treated with amoxicillin at a dose of 89 mg/kg (*p.o*) for 8 days to prevent local infection. Weights were measured on weekly basis and the animals were palpated twice a week in order to detect possible mammary tumor. The time of tumor onset was noted. Autopsy was performed on animals that died during the experiment. At the end of the experiment all the survivors were subjected to a 12-h fast and then sacrificed under anesthesia (10 mg/kg ketamine and 50 mg/kg valium) by decapitation. Blood was collected into EDTA tubes by intracardiac puncture for hematological analyzes on the one hand and in the dry tubes to obtain serum for assessment of cytokine levels (TNF-α, IL -6, IL-12, IFN-γ) and some toxicity markers (ALT and creatinine). In addition, mammary tumors and some toxicity organs (liver, kidneys, lungs, spleen, adrenals, thymus, uterus, ovaries and heart) were collected and maintained in 4% formol for histological analysis. Tumor volume, incidence and tumor burden were calculated using the respective formulas length × width × height × π/6, rat with tumors/total number of rats ×100 and tumor mass/animal mass (Faustino-Rocha et al., 2013). The antioxidant markers (SOD, CAT, MDA, NO) and total proteins was measured in a 20% homogenate of the mammary gland and breast tumor.

#### 2.7.4 Histopathological analysis

Histopathological analysis of mammary tumors was done at the Department of Morphological Sciences and Pathological Anatomy, Faculty of medicine and Biomedical Sciences in the University of Garoua (Cameroon) following Russo and Russo (2000) criteria with basic histological techniques - trimming, dehydration and inclusion. Hematoxylin and eosin stains were used for 5-µm paraffin embedded tissues from collected organs and tumors. Photomicrographs were obtained using a light Axioskop 40 microscope connected to a computer and analysis of images was carried out with ImageJ software at magnification of ×200.

#### 2.7.5 Quantification of inflammatory cytokine levels

The inflammatory cytokine levels notably interferon gamma (IFN-γ), tumor necrosis factor-alpha (TNF-α), epidermal growth factor (EGF), interleukin-6 (IL-6), interleukin-12 (IL-12) and fractalkine were measured with magnetic luminex screening assay described by Khan et al. (2004). Rat premixed MILIPLEX® Kit (Millipore, Minneapolis, United States) was used to perform the assay according to the instructions of the manufacturer. Briefly, after a dilution of samples, 50 µL/well of standard cytokines were placed in 96-well microplates in a horizontal manner. In the same way, 50 µL of rat, magnetic, premixed, microparticle cocktail with antibodies specific for each cytokine. Washing and incubation of microplates was done using a magnetic plate separator and a 50 µL/well cocktail of biotin-antibodies specific for each cytokine. Thereafter, antibody-cytokine complexes were detected by Streptavidin-PE and the analysis done inside a luminex MAGPix Analyzer (XMAP Technology, SN, United States). Results were expressed as median fluorescence intensity (MFI). Lastly, the conversion of MFI in cytokine relative concentration was carried out through a standard curve specific to each cytokine. The limit values for quantification of cytokine in pg/mL were 5.2 (IFN- γ), 0.3 (EGF), 1.9 (TNF-α), 0.2 (IL-6), 0.2 (fractalkine), 0.4 (IL-12), were the lowest level of detectable concentration for cytokines.

#### 2.7.6 Oxidative stress markers quantification

Antioxidant makers - superoxide dismutase (SOD) activity (IU) (Christodoulou et al., 2022), catalase (CAT) activity (mM/min/mg) (Hadwan and Abed, 2016) and oxidative stress markers- Malondialdehyde (MDA) level (µmol/g proteins) (De Leon and Borges, 2020) and nitric oxide (NO) level (µM) (Bryan and Grisham, 2007) in mammary glands and tumors were determined. Total protein levels of sample were also determined for expression of maker level results using biuret reaction described by Straub et al. (2023).

#### 2.7.7 Blood analysis

Dry tubes with collected blood were centrifuged at 3000 rpm, 5 °C for 15 min to obtain serum which was stored at −20 °C for measurement of biochemical makers. Reagent kits (Fortress Diagnostics Limited, Muckamore, United Kingdom) was used to measure alanine transaminase (ALT) activity (IU/L) and creatinine level (mg/dl) following Silihe et al. (2023).

Hematological parameters were assessed following Chhabra (2018) with a HumaCount 30 T Automated Hematology Analyzer (Human Diagnostics Worldwide, Wiesbaden, Germany) – white blood cell (WBC) count (×10^3^µL^−1^), monocytes (%), granulocytes (%), lymphocytes (%), red blood cell (RBC) count (×10^3^µL^−1^), hemoglobin (g/dL), hematocrit (%), mean corpuscular hemoglobin or MCH (pg), mean corpuscular hemoglobin concentration or MCHC (g/dL), mean corpuscular volume or MCV (fL) and platelets count (×10^3^µL^−1^).

### 2.8 Statistical analysis

Results of the *in vivo* experimental groups and three independent *in vitro* assays carried out in triplicate were expressed as mean ± standard error of mean (SEM). Statistical analysis of data was done using GraphPad Prism software version 5.00. One-way analysis of variance (ANOVA) followed by Dunnett’s *post hoc* test for multiple comparisons was done. Survival was calculated by the Kaplan-Meier method using GraphPad Prism software, and hazard ratios were calculated using Mantel-Cox proportional hazard regression with 95% confidence intervals. The probability level *p* < 0.05 was considered as statistical significance of differences.

## 3 Results

### 3.1 *LC-MS analysis of the polyphenol-enriched fraction of Acacia seyal* Delile

**FIGURE 1:**
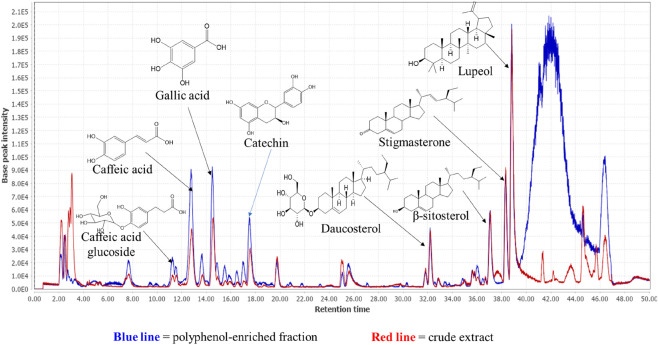
Base peak chromatogram of crude *Acacia seyal* crude extract (read line) and its derived phenolic fraction (blue line) obtained by UHPLC-HR-LC-MS.

The comparative UHPLC-HR-LC-MS chromatograms of polyphenol-enriched fraction (ASpo) and crude extract (ASHE) of *A. seyal* (Figure 1), shows that they are different and contain a relative abundance of compounds. The compounds such as caffeic acid, gallic acid, catechin, daucosterol, β-sitosterol, stigmasterone and lupeol have been putatively detected (Table 1) (Ashour et al., 2022).

**TABLE 1:** Summary of compounds separated and identified in *Acacia seyal* Delile phenolic fraction by UHPLC-ESI-HRMS analysis in the negative ion mode.

Pick N°	Tr (min)	[M-H]^-^(*m/z*)	EC	Mass errors (ppm)	RDBeq	Fragment ions (m/z)	Tentative identification
1	12.10	343.08	C_14_H_15_O_8_	0.3	7.5	137.02	Caffeic acid 4-O-glucoside
2	13.20	179.09	C_15_H_17_O_9_	1.5	7.5	167.03	Caffeic acid
3	13.80	353.09	C_16_H_17_O_9_	1.4	8.5	191.06	Caffeoylquinic acid
4	14.81	169.04	C_9_H_7_O_3_	−5.5	6.5	​	Gallic acid
5	66.26	165.05	C_9_H_9_O_3_	−5.5	5.5	​	Phloretic acid
6	32.332	575.8	C_35_H_6_O_6_	−2.5	6.5	431.31	Daucosterol
7	37.36	413.34	C_29_H_50_O	0.4	7.5	487.34	β-Sytosterol
8	38.22	413.34	C_30_H_47_O_5_	0.2	7.5	​	Triterpenoid
9	38.503	413.33	C_29_H_48_O	1.7	7.5	411.33	Stimasterol
10	39.7	425.73	C_30_H_50_O	−0.6	8.5	415.32	Lupeol
11	13.82	487.34	C_30_H_47_O_5_	−0.8	7.5	425.35	Triterpenoid
12	14.399	485.33	C_30_H_45_O_5_	2.3	8.5	471.35	Triterpenoid
13	14.57	487.34	C_30_H_47_O_5_	0.8	7.5	459.35	Triterpenoid

Tr, Retention Time; [M-H]-, Molecule minus one proton, with negative charge; EC, Elemental Composition; RDBeq, Ring Double Bond Equivalent.

### 3.2 Cell growth, cell proliferation, clone formation and caspase 3 activity

#### 3.2.1 Cell growth and proliferation

**FIGURE 2:**
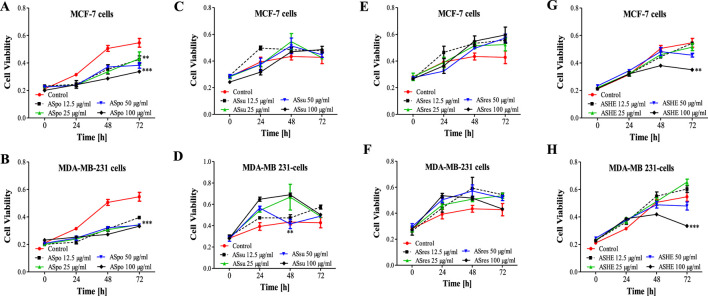
Growth of estrogen-sensitive MCF-7 and triple negative MDA-MB-231 breast carcinoma cells treated with different concentrations of polyphenol-enriched-ASpo **(A,B)**, polysaccharide-ASsu **(C,D)** and residue-ASres **(E,F)** fractions from the *Acacia seyal* hydro-ethanolic crude extract-ASHE **(G,H)** after 24, 48 and 72 h **p < 0.01 and ***p < 0.001 compared to control.

ASsu and ASres did not inhibit MCF-7 and MDA-MB-231 cell growth (Figures 2C–F), while a significant reduction (*p* < 0.01 and *p* < 0.001) of this cell growth was observed with ASpo after 48 and 72 h at 25, 50 and 100 μg/mL concentrations (Figures 2A,B). The crude extract (ASHE) exhibited such effect only at 100 μg/mL concentration in MCF-7 (*p* < 0.01) and MDA-MB-231 cells (Figures 2G,H).

**FIGURE 3:**
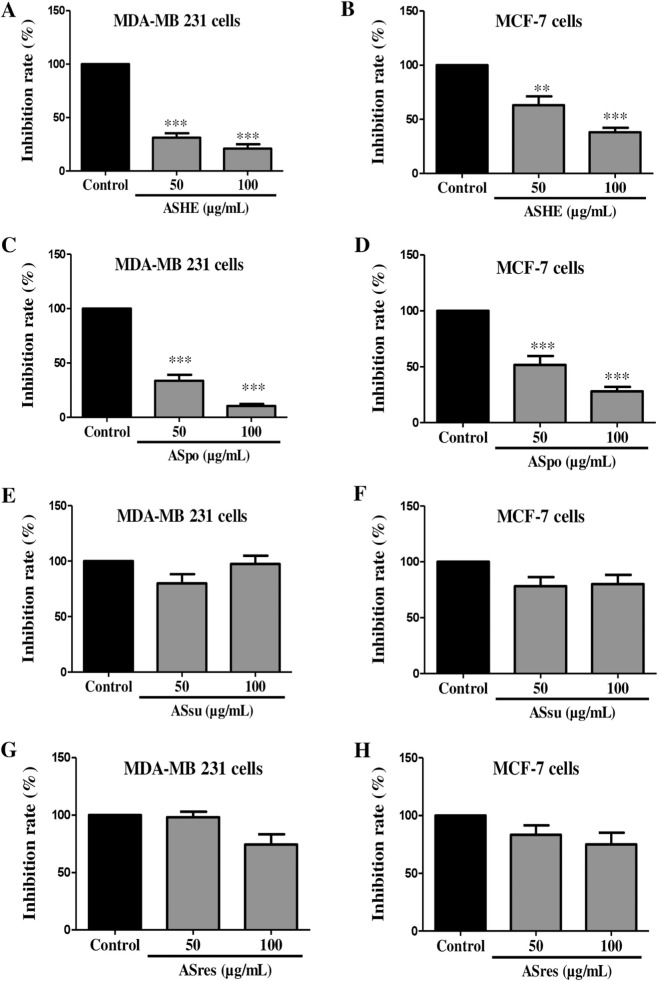
Cell proliferation of triple negative MDA-MB-231 and estrogen-sensitive MCF-7 breast carcinoma cells treated with *Acacia seyal* hydro-ethanolic extract-ASHE **(A,B)**, and polyphenol-enriched fraction -ASpo **(C,D)**, polysaccharide-ASsu **(E,F)** and residue - ASres **(G,H)** fractions after 48 h. Controls remained untreated. (n = 3). **p < 0.01 and ***p < 0.001 compared to control.

Only ASpo and ASHE reduced (*p* < 0.001) MDA-MB-231 (Figures 3A,C) and MCF-7 (Figures 3B,D) cell proliferation at 50 and 100 μg/mL in a concentration-dependent manner.

#### 3.2.2 *Clone formation* and caspase 3 activity

**FIGURE 4:**
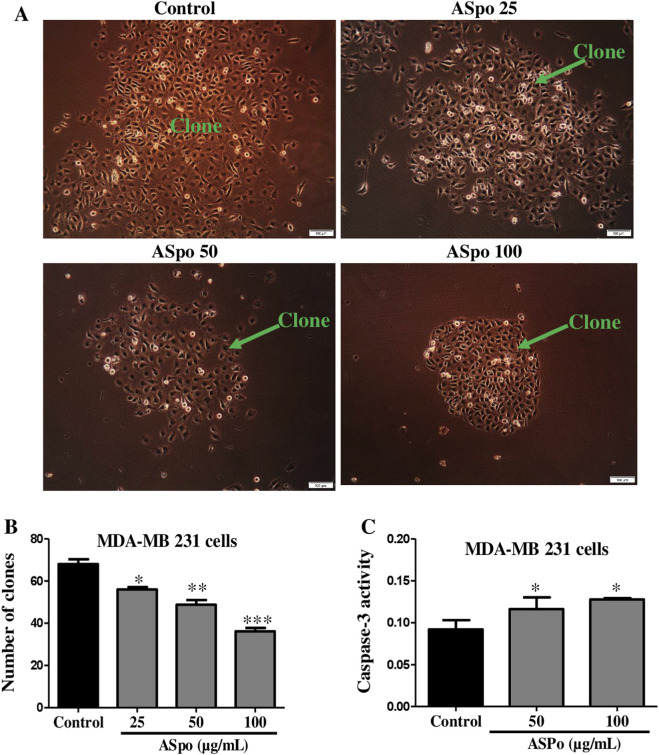
Inhibitory effects of *Acacia seyal* polyphenol-enriched (ASpo) fraction on MDA-MB 231 clone formation **(A,B)** after 7 days. Caspase-3 **(C)** assay after 48 h of incubation of MDA-MB-231 breast carcinoma cells with ASpo. Controls remained untreated (n = 3). Treated cancer cell cultures were compared to non-treated control cultures of the same passage and cell numbers per well. *p < 0.05, **p < 0.01 and ***p < 0.001 compared to control.

ASpo significantly inhibited the number of clones at the tested concentrations of 25 (*p* < 0.05), 50 (*p* < 0.01) and 100 μg/mL (*p* < 0.001) following a concentration-dependent shape compared to untreated cells (Figures 4A,B). The most potent effect was observed at 100 μg/mL concentration.

ASpo incubated with MDA-MB-231 cells for 48 h, increased (*p* < 0.05) caspase-3 activity (Figure 4C) at the concentrations of 50 and 100 μg/mL compared to control (untreated cells).

### 3.3 Anti-metastatic potential of polyphenol-enriched fraction

#### 3.3.1 Cell migration and invasion

**FIGURE 5:**
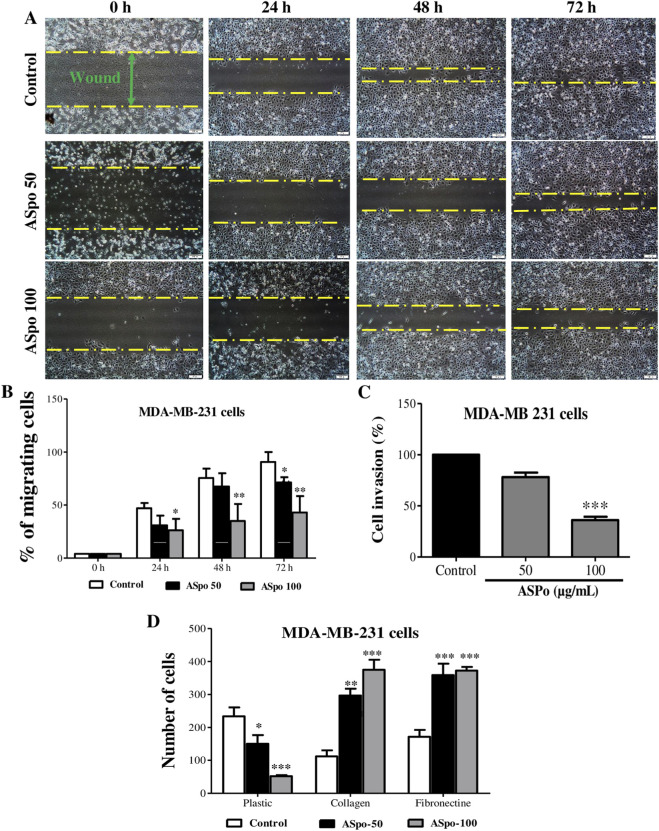
Effects of *Acacia seyal* polyphenol-enriched fraction (ASpo) on triple negative MDA-MB-231 cell migration, cell invasion and adhesion. Microphotographs of one assay **(A)** and graphic representation of three independent wound healing assays **(B)** in MDA-MB-231 cell migration after 72 h. Controls remained untreated. (n = 3). Cell invasion **(C)** and cell adhesion **(D)**. Treated cancer cell cultures were compared to non-treated control cultures of the same passage and cell numbers per well. *p < 0.05 and **p < 0.01, ***p < 0.001 as compared with control.

ASpo (50 and 100 μg/mL) inhibited MDA-MB-231 cell migration toward cell-free space of microphotographs (Figure 5A) in a significant way after 24 h (*p* < 0.05 at 100 μg/mL), 48 h (*p* < 0.01 at 100 μg/mL) and 72 h (*p* < 0.05 at 50 μg/mL and *p* < 0.01 at 100 μg/mL) compared to control cells (Figure 5B).

ASpo at the high concentration of 100 μg/mL reduced (*p* < 0.001) MDA-MB-231 cells invasion through the transwell membrane compared to control cells (Figure 5C).

#### 3.3.2 Cell adhesion to the extracellular matrix components

The effects of ASpo on cell adhesion matrix components are shown in Figure 5D. ASpo significantly increased cell adhesion in collagen (*p* < 0.01 at 50 and *p* < 0.001 at 100 μg/mL) and fibronectin (*p* < 0.001 at both 50 at 100 μg/mL) extracellular matrix component when compared to control. While cell adhesion reduced (*p* < 0.05 at 50 and *p* < 0.001 at 100 μg/mL) in the cells treated with ASpo in plastic.

### 3.4 Chemopreventive effects of A. seyal polyphenol-enriched fraction

#### 3.4.1 Body weight and survival rate

**FIGURE 6:**
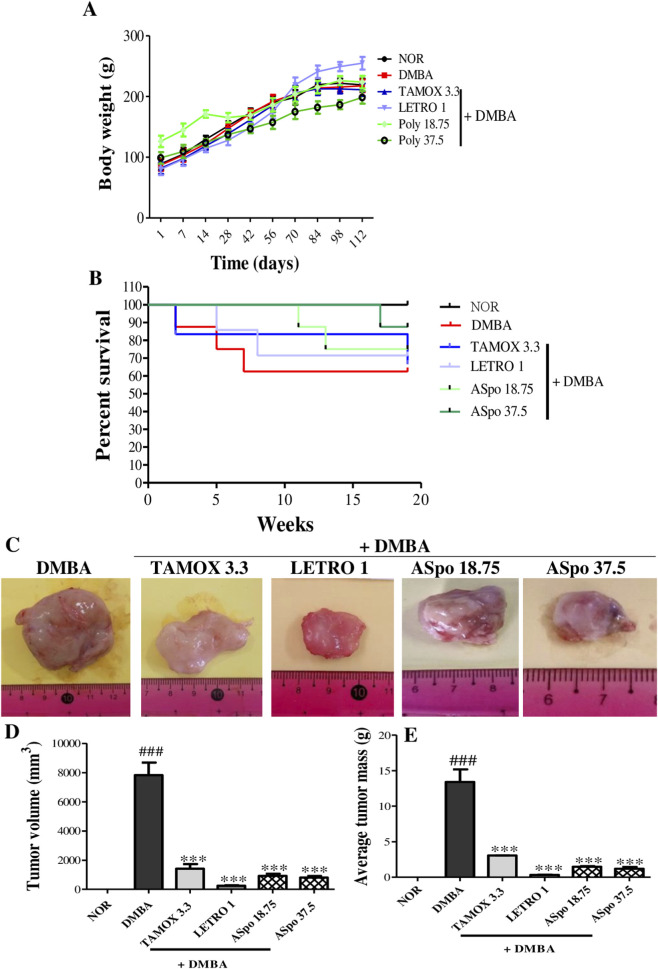
Effects of *Acacia seyal* polyphenol-enriched fraction (ASpo) on body weight **(A)**, Kaplan Meir curve of survival **(B)**, tumor morphology **(C)**, tumor volume **(D)** and tumor mass **(E)**. NOR, normal control rats receiving distilled water; DMBA, rats serving as negative control and receiving vehicle; TAMOX 3.3 + DMBA rats serving as positive control 1 and receiving tamoxifen at the dose of 3.3 mg/kg; LETRO 1 + DMBA, rats serving as positive control two and receiving letrozole at the dose of 1 mg/kg; ASpo 18.75 + DMBA and ASpo 37.5 + DMBA, rats receiving polyphenol-enriched extract of *A. seyal* at the dose of 18.75 mg/kg and 37.5 mg/kg. All animals except those in the normal group (NOR) were injected with a single dose of DMBA (50 mg/kg). Significance compared to the NOR group: ###*p <* 0.001. Significance compared to the DMBA group: ***p < 0.001.

Figure 6A shows body weight evolution of animals after 20-week experiment. No significant change in body weight was observed in the different experimental groups.

The Kaplan-Meier curve (Figure 6B) presents the survival rate following 20-week experiment. No rat from the normal group (NOR) died during the experiment. In the DMBA group, there were 3 deaths out of 8 animals (62.5% survival rate; HR: 1.211, 95% CI: 1.2–7.3, p < 0.05). Rats receiving tamoxifen and letrozole each had 2 deaths out of 8 (75% survival rate), similar to rats receiving ASpo at the lowest dose of 18.75 mg/kg. One death out of 8, representing the lowest incidence of mortality (87.5% survival rate; HR: 0.711, 95% CI: 1.5–5.8, *p* < 0.05), was observed with the higher dose of ASpo (37.5 mg/kg).

#### 3.4.2 Tumor parameters and morphology

At the end of 20 weeks of experiment (Table 2) DMBA group presented 100% mammary tumor incidence (8/8 animals with tumor) and 97.28 ± 31.32 g/kg of tumor burden compared to normal group. ASpo comparable to reference drugs (letrozole and tamoxifen) reduced tumor incidence of 37.5% (3/8 animals with tumor) at 18.75 mg/kg and 50% (4/8 animals with tumor) at 37.5 mg/kg compared to DMBA group. ASpo also reduced tumor burden of 17.81 ± 6.09 g/kg (*p* < 0.01) at 18.75 mg/kg (82% of inhibition) and 7.44 ± 2.15 g/kg *(p* < 0.001) at 37.5 mg/kg (92% of inhibition) compared to DMBA group. Similarly to reference drugs, ASpo as show on tumor photographs (Figure 6C) at all tested doses significantly (*p* < 0.001) reduced average tumor mass (Figure 6E) and mammary tumor volume (Figure 6D) at 924.55 ± 134.19 mm^3^ (18.75 mg/kg) and 804.32 ± 106.96 mm^3^ (37.5 mg/kg) compared to 7827.30 ± 856.46 mm^3^ (DMBA group).

**TABLE 2:** Effects of Acacia seyal polyphenol-enriched fraction on tumor incidence and tumor burden.

	Tumor incidence	Tumor burden (g/kg)	% Inhibition of tumor burden
NOR	0% (0/8)	-	-
DMBA	100% (8/8)	97.28 ± 31.32###	-
TAMOX 3.3 + DMBA	25% (2/8)	15.20 ± 0.03***	84%
LETRO 1 + DMBA	37.5% (3/8)	3.72 ± 1.35***	96%
ASPo 18.75 + DMBA	37.5% (3/8)	17.81 ± 6.09**	82%
ASpo 37.5 + DMBA	50% (4/8)	7.44 ± 2.15***	92%

NOR, normal control rats receiving distilled water ; DMBA, rats serving as negative control and receiving vehicle; TAMOX 3.3 + DMBA rats serving as positive control 1 and receiving tamoxifen at the dose of 3.3 mg/kg ; LETRO 1 + DMBA, rats serving as positive control 2 and receiving letrozole at the dose of 1 mg/kg ; ASpo 18.75 + DMBA and ASpo 37.5 + DMBA, rats receiving polyphenol-enriched extract of A. seyal at the dose of 18.75 mg/kg and 37.5 mg/kg. All animals except those in the normal group (NOR) were injected with a single dose of DMBA (50 mg/kg). Significance compared to the NOR group: ###p < 0.001. Significance compared to the DMBA group: **p < 0.01, ***p < 0.001.

#### 3.4.3 Histopathological analysis

**FIGURE 7:**
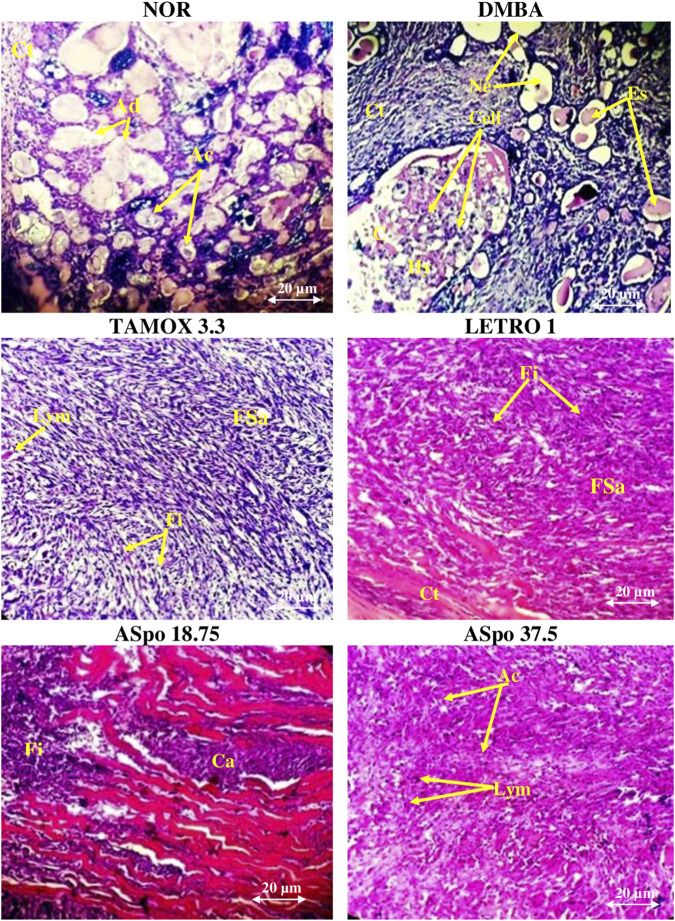
Histopathology of mammary glands and mammary tumors following *Acacia seyal* polyphenol-enriched fraction (ASpo) treatment. NOR, normal control rats receiving distilled water; DMBA, rats serving as negative control and receiving vehicle; TAMOX 3.3 + DMBA rats serving as positive control 1 and receiving tamoxifen at the dose of 3.3 mg/kg; LETRO 1 + DMBA, rats serving as positive control two and receiving letrozole at the dose of 1 mg/kg; ASpo 18.75 + DMBA and ASpo 37.5 + DMBA, rats receiving polyphenol-enriched extract of *A. seyal* at the dose of 18.75 mg/kg and 37.5 mg/kg. All animals except those in the normal group (NOR) were injected with a single dose of DMBA (50 mg/kg). Ac = dilated acinus ducts; Ad = Adipose tissue; Ct = Conjonctive tissue; Hy = typical hyperplasia with dilated ducts; Cell = macrophage with cellular debris; Lym = Lymphocyte; Fsa = Fibrosarcoma; Fi = young fibrobast; Es = Eosinophile secretion; Ca = cribriform carcinoma.

The mammary gland of normal rats stained by hematoxylin (H) and eosin (E) presented normal acinus without infiltration and important adipocytes (Figure 7). Histological analysis of rats treated only with DMBA revealed an advanced grade SBRIII of a cribriform ductal carcinoma with poor lymphocyte infiltration (10%) and comedonecrosis (30%). Rats that received the reference drug, tamoxifen or letrozole both showed a fibrosarcoma with a low-grade SBRI, 10% of comedonecrosis and no lymphocyte infiltration. Mammary tumors of rats which received ASpo were associated to a middle grade SBRII and a high level of lymphocyte infiltration at the value of 30% for high dose 37.5 mg/kg BW with ductal carcinoma and a fibrosarcoma with 35% of comedonecrosis for the low dose 18.75 mg/kg BW.

#### 3.4.4 Inflammatory cytokines

**FIGURE 8:**
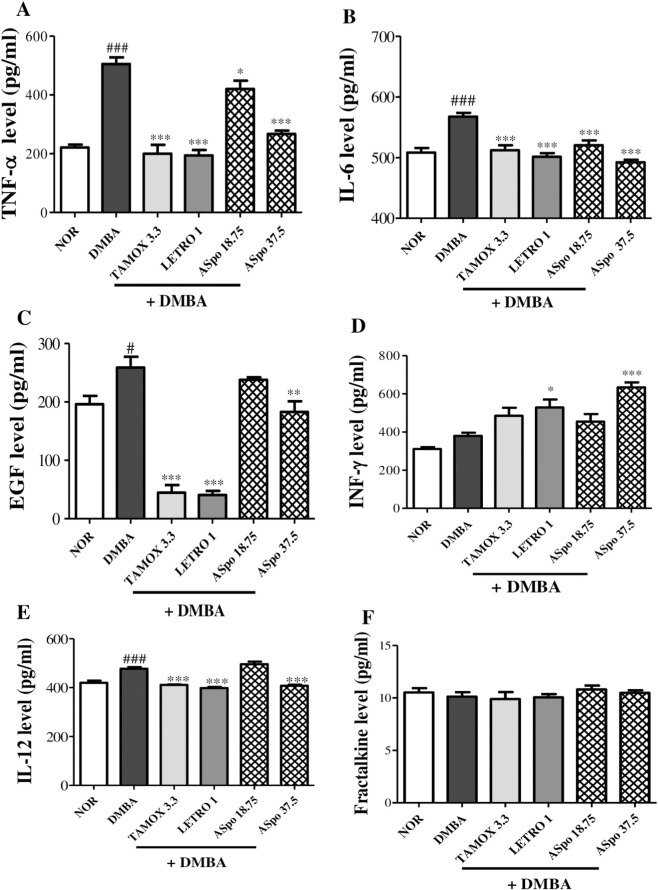
Effects of *Acacia seyal* polyphenol-enriched fraction (ASpo) on TNF-α **(A)**, IL-6 **(B)**, EGF **(C)**, IFN-γ **(D)**, IL-12 **(E)** and Fractalkine level **(F)**. NOR, normal control rats receiving distilled water; DMBA, rats serving as negative control and receiving vehicle; TAMOX 3.3 + DMBA rats serving as positive control 1 and receiving tamoxifen at the dose of 3.3 mg/kg; LETRO 1 + DMBA, rats serving as positive control two and receiving letrozole at the dose of 1 mg/kg; ASpo 18.75 + DMBA and ASpo 37.5 + DMBA, rats receiving polyphenol-enriched extract of *A. seyal* at the dose of 18.75 mg/kg and 37.5 mg/kg. All animals except those in the normal group (NOR) were injected with a single dose of DMBA (50 mg/kg). Significance compared to the NOR group: #*p <* 0.05, ###*p <* 0.001. Significance compared to the DMBA group: *p < 0.05, **p < 0.01, ***p < 0.001.

Figure 8 presents the effects of 20-week treatment with reference drugs (letrozole and tamoxifen) and ASpo on inflammatory cytokine levels. ASpo at low dose (18.75 mg/kg) decreased TNF-α (*p* < 0.05) and IL-6 (*p* < 0.001) levels only while the higher dose (37.5 mg/kg) significantly (*p* < 0.001) decreased TNF-α, IL-6, IL-12 levels comparable to reference drugs and EGF (*p* < 0.01) level inferior to reference drugs compared to DMBA group. This higher dose (37.5 mg/kg) of ASpo (*p* < 0.001) superior to letrozole (*p* < 0.05) significantly increased IFN-γ level compared to DMBA group. No significant variation of fractalkine level was recorded in the different groups.

#### 3.4.5 Oxidative stress and antioxidant markers

**FIGURE 9:**
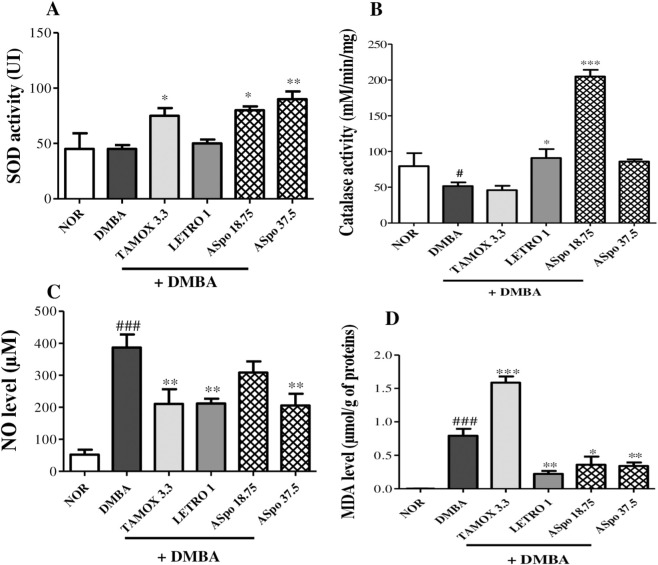
Effects of *Acacia seyal* polyphenol-enriched (ASpo) fraction on SOD **(A)** and catalase **(B)** activities as well as NO **(C)** and MDA **(D)** levels. NOR, normal control rats receiving distilled water; DMBA, rats serving as negative control and receiving vehicle; TAMOX 3.3 + DMBA rats serving as positive control 1 and receiving tamoxifen at the dose of 3.3 mg/kg; LETRO 1 + DMBA, rats serving as positive control 2 and receiving letrozole at the dose of 1 mg/kg; ASpo 18.75 + DMBA and ASpo 37.5 + DMBA, rats receiving polyphenol-enriched extract of *A. seyal* at the dose of 18.75 mg/kg and 37.5 mg/kg. All animals except those in the normal group (NOR) were injected with a single dose of DMBA (50 mg/kg). Significance compared to the NOR group: #*p <* 0.05, ###*p <* 0.001. Significance compared to the DMBA group: *p < 0.05, **p < 0.01, ***p < 0.001.

The results of oxidative stress and antioxidant markers after treatment with ASpo are depicted on Figure 9. Superior to tamoxifen, ASpo mainly at 37.5 mg/kg (*p* < 0.01) increased SOD activity and at low dose (18.75 mg/kg) superior to letrozole increased (*p* < 0.001) catalase activity compared to DMBA group. At the higher dose (37.5 mg/kg) comparable to tamoxifen, ASpo reduced (*p* < 0.01) NO level and in dose dependent manner (18.75 mg/kg, *p* < 0.05 and 37.5 mg/kg, *p* < 0.01) similarly to letrozole reduced MDA level compared to DMBA group.

### 3.5 Toxicological profile of Acacia seyal polyphenol-enriched fraction

#### 3.5.1 Hematological parameters

The treatments with different substances on hematological parameters are showed in Table 3. ASpo at all tested doses (*p* < 0.05) comparable to reference drugs (*p* < 0.001) increased lymphocyte count, platelets and hemoglobin and decreased (*p* < 0.001) percentage of granulocytes compared to DMBA group. The higher dose of ASpo (37.5 mg/kg, *p* < 0.01) inferior to letrozole (*p* < 0.001) increased mean corpuscular hemoglobin concentration (MCHC) compared to DMBA group.

**TABLE 3:** Effects of Acacia seyal polyphenol-enriched fraction on hematological parameters.

Parameters	NOR	+ DMBA				
		DMBA	TAMOX 3.3	LETRO 1	ASpo 18.75	ASpo 37.5
WBC (×10^3^µL^−1^)	15.422 ± 0.58	12.457 ± 0.62	13.5 ± 1.10	6.944 ± 0.64**	10.56 ±1.87	12.88 ± 1.47
Lymphocytes (%)	74.11 ± 2.82	66 ± 0.44#	79.72 ± 2.48***	83.56 ± 1.19***	74.68 ± 1.70*	74.10 ± 1.82*
Monocytes (%)	5.2 ± 0.547	6.32 ± 0.901	4.65 ± 0.96	3.824 ± 0.69	10.72 ± 2.82*	15.42 ± 1.95***
Granulocytes (%)	18.4 ± 1.74	27.62 ± 1.34###	16.8 ± 1.38***	13.2 ± 0.63***	14.60 ± 0.42***	16.3 ± 1.76***
RBC (×10^3^µL^−1^)	8 ± 0.53	6.22 ± 0.27	7.84 ± 0.43	7.47 ± 0.76	8.19 ± 0.15*	7.21 ± 0.65
Hematocrit (%)	44.2 ± 1.02	40.45 ± 2.39	50.66 ± 2.10*	46.46 ± 2.89	50.98 ± 1.60**	45.14 ± 4.11
MCV (fL)	62.45 ± 1.51	64.6 ± 0.93	66.2 ± 0.47	56.92 ± 0.80**	64.12 ± 0.75	64.74 ± 2.69
Platelets (×10^3^µL^−1^)	379.0 ± 0.6	291.5 ± 6.75###	892.5 ± 25.14***	627 ± 23.08***	735.72 ± 63.05***	621.5 ± 68.94***
MCH (pg)	17.225 ± 0.256	17.55 ± 0.663	16.74 ± 0.332	17.64 ± 0.235	17.38 ± 0.32	18.8 ± 0.28
Hémoglobin (g/dL)	12.3 ± 0.18	7.925 ± 0.31###	13.1 ± 0.45***	14.22 ± 0.68***	13.88 ± 0.33***	13.51 ± 1.16***
MCHC (g/dL)	27.6 ± 0.24	26.15 ± 0.35	26.34 ± 0.11	31 ± 0.67***	27.28 ± 0.30	29.16 ± 0.96**

NOR, normal control rats receiving distilled water ; DMBA, rats serving as negative control and receiving vehicle; TAMOX 3.3 + DMBA, rats serving as positive control 1 and receiving tamoxifen at the dose of 3.3 mg/kg ; LETRO 1 + DMBA, rats serving as positive control 2 and receiving letrozole at the dose of 1 mg/kg ; ASpo 18.75 + DMBA and ASpo 37.5 + DMBA, rats receiving Acacia seyal polyphenol-enriched fraction at the dose of 18.75 mg/kg and 37.5 mg/kg. All animals except those in the normal group (NOR) were injected with a single dose of DMBA (50 mg/kg). Data are represented as mean ± SEM (n=8). # p < 0.05; ### p < 0.001 compared to NOR ; * p < 0,05 ; ** p < 0,01 and *** p < 0,001 compared to DMBA group. WBC = White blood cells; RBC =Red blood cells; MCV = Mean Corpuscular Volume; MCH = Mean Corpuscular Hemoglobine; MCHC = Mean Corpuscular Hemoglobin Concentration.

#### 3.5.2 Relative mass of different organs, creatinine and ALT activity

The effects of ASpo on toxicity organs, creatinine and alanine transaminase (ALT) activity are presented by Table 4. The rats that only received DMBA presented a significant (*p* < 0.01) increase in relative mass of uterus, liver, adrenals gland and spleen compared to normal group. ASpo at all tested doses (18.75 and 37.5 mg/kg) remarkably (*p* < 0.001) restored toward normal relative mass of thymus, liver, adrenals, femur and spleen similarly to reference drugs as compared to DMBA group. While these tested doses (18.75 and 37.5 mg/kg) restored toward normal relative mass of heart in a manner superior to reference drugs as compared to DMBA group. The lower dose of ASpo (18.75 mg/kg) inferior to reference drugs (*p* < 0.001) decreased (*p* < 0.05) the relative mass of uterus compared to DMBA group. ASpo only at the higher dose (37.5 mg/kg) superior to tamoxifen (*p* < 0.05) and comparable to letrozole (*p* < 0.001) increased relative mass of ovaries compared to DMBA group.

**TABLE 4:** Effects of *Acacia seyal* polyphenol-enriched fraction on relative mass of different organs, kidney and liver toxicity markers.

Items	NOR	+ DMBA				
		DMBA	TAMOX 3.3	LETRO 1	ASpo 18.75	ASpo 37.5
Relative mass of organs (mg/kg)						
Thymus	1396.65 ± 40.29	767.96 ± 40.32###	1423.66 ± 20.88***	976.36 ± 42.97**	1353.69 ± 56.77***	1309.79 ± 51.53***
Uterus	1575.7 ± 70.79	2078.78 ± 153.80##	1175.43 ± 10.01***	607.74 ± 20.05***	1667.29 ± 110.14*	1807.32 ± 147.81
Liver	30380.97 ± 739.11	34213.32 ± 1489.89##	30922.61 ± 622.77**	29049.94 ± 707.68***	29873.69 ± 865.87***	27790.56 ± 1043.64***
Kidneys	5617.89 ± 113.58	5780.87 ± 322.25	6402.08 ± 83.62	4851.48 ± 103.13**	5281.21 ± 162.46	5106.05 ± 280.79
Adrenals	343.32 ± 13.19	455.99 ± 38.98##	406.49 ± 3.58	157.3 ± 3.39***	246.6 ± 13.68***	303.11 ± 24.91***
Lungs	6606.89 ± 217.61	7082.42 ± 228.57	5886.97 ± 49.19**	5381.24 ± 139.62***	7056.65 ± 363.06	6539.26 ± 263.73
Femur	2569.67 ± 84.66	2256.91 ± 105.97	3044.18 ± 41.15***	2647.83 ± 90.96	2845.18 ± 142.86**	3177.78 ± 134.56***
Spleen	5999.93 ± 85.47	7316.01 ± 523.67##	4335.26 ± 44.53***	3055.72 ± 19.89***	3745.85 ± 116.58***	3831.38 ± 198.92***
Ovaries	550.61 ± 10.64	531.82 ± 25.01	438.93 ± 5.83*	777.16 ± 20.86***	541.75 ± 26.66	685.3 ± 39.25***
Heart	3107.36 ± 64.82	2621.4 ± 149.12###	2666.14 ± 33.76	2711.34 ± 34.76	3164.06 ± 66.94***	3106.85 ± 74.69***
Kidney and liver toxicity markers						
ALT (IU/L)	193.66 ± 28.29	259.00 ± 25.76	259.29 ± 36.87	283.33 ± 45.43	216.41 ± 44.62	256.8 ± 33.21
Creatinine (mg/dL)	0.73 ± 0.03	1.82 ± 0.21###	0.78 ± 0.12***	0.65 ± 0.04***	0.84 ± 0.13***	0.93 ± 0.07**

NOR, normal control rats receiving distilled water; DMBA, rats serving as negative control and receiving vehicle; TAMOX, 3.3 + DMBA, rats serving as positive control 1 and receiving tamoxifen at the dose of 3.3 mg/kg; LETRO, 1 + DMBA, rats serving as positive control 2 and receiving letrozole at the dose of 1 mg/kg; ASpo, 18.75 + DMBA, and ASpo, 37.5 + DMBA, rats receiving polyphenol-enriched extract of *A. seyal* at the dose of 18.75 mg/kg and 37.5 mg/kg. All animals except those in the normal group (NOR) were injected with a single dose of DMBA (50 mg/kg). Data are represented as mean ± SEM (n = 8). ##*p* < 0.01; ###*p* < 0.001 compared to NOR; *p < 0,05; **p < 0,01; ***p < 0.001 compared to DMBA, group.

On biochemical markers of toxicity, ASpo at all tested doses (18.75 and 37.5 mg/kg) similarly to reference drugs reduced creatinine level in dose dependent manner (*p* < 0.01) compared to DMBA group. No significant change was observed in alanine transaminase activity in different groups.

#### 3.5.3 Histo-architecture of some toxicity organs

**FIGURE 10:**
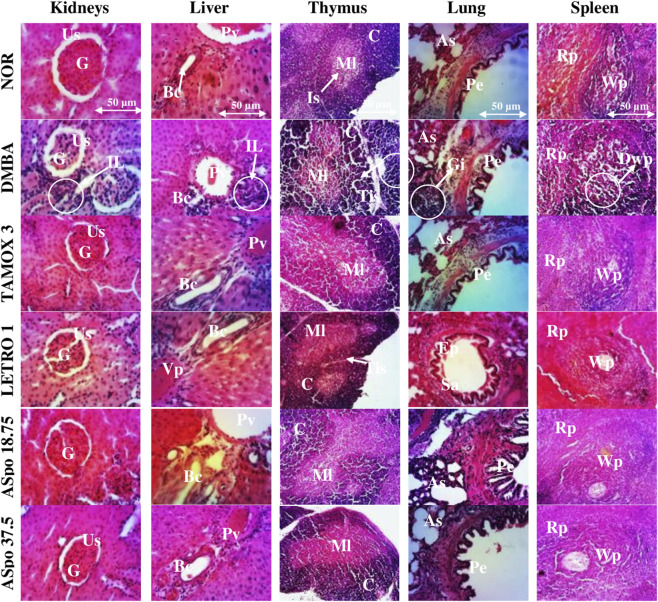
Effects of *Acacia seyal* polyphenol-enriched fraction on histoarchitecture of some organs of interest in toxicity. NOR, normal control rats receiving distilled water; DMBA, rats serving as negative control and receiving vehicle; TAMOX 3.3 + DMBA rats serving as positive control 1 and receiving tamoxifen at the dose of 3.3 mg/kg; LETRO 1 + DMBA, rats serving as positive control 2 and receiving letrozole at the dose of 1 mg/kg; ASpo 18.75 + DMBA and ASpo 37.5 + DMBA, rats receiving polyphenol-enriched extract of *A. seyal* at the dose of 18.75 mg/kg and 37.5 mg/kg. All animals except those in the normal group (NOR) were injected with a single dose of DMBA (50 mg/kg). Kidneys: Us = Urinary space, G = Glomerulus, Li = Leukocyte infiltration, Liver: Pv = Portal vein, Bc = Bile canaliculus, Li = Leukocyte infiltration, Thymus: Ml = Medullary layer, Is = Interlobular septum, C = Cortex, Tis = Thickening of the interlobular septum; Lung: Pe = Pulmonary epithelium, As = Alveolar sac, Gi = Inflammatory granuloma; Spleen: Wp = White pulp, Rp = Red pulp, Dwp = Disorganization of the white pulp.

Figure 10 shows the effects of ASpo on the microarchitecture of the kidney, spleen, liver, lung and thymus. DMBA group showed a leukocyte infiltration in kidneys, liver, lungs, a disorganization of the white pulp in spleen and a decrease in the density of cortical lymphocytes with thickening of the interlobular septum compared to normal group. This normal group exhibited normal histological architecture in the liver, lungs, kidneys (well-differentiated urinary space and glomerulus) and spleen (well-differentiated white and red pulp). ASpo at all tested doses (18.75 and 37.5 mg/kg) similarly to reference drugs showed an important thymus cortical lymphocyte density and a differentiated renal, hepatic and pulmonary histoarchitecture without major alterations compared to DMBA group.

## 4 Discussion

Previous studies on the hydro-ethanolic extract of *A. seyal* stem barks showed that this extract has an *in vitro* cytotoxicity on estrogen-dependent MCF-7 and triple-negative MDA-MB-231 cells, due to its ability to trigger intrinsic apoptosis and inhibit cell migration and invasion (Zingue et al., 2018a). In addition, it prevented DMBA-induced mammary tumors in Wistar rats (Zingue et al., 2018b). Polyphenols from certain enriched extracts of European blueberry, Chinese tea, American pecan nutshells and Korean *N. nucifera* were reported to have significant anticancer activities against breast cancer, in the same way as non-toxic macromolecules (polysaccharides) present in natural products with immunomodulatory properties (Li et al., 2016; Vuong et al., 2016; Ribas et al., 2023). The present study was therefore conducted to improve the bioactivity of *A. seyal* hydro-ethanolic extract (ASHE) following its fractionation in polyphenol-enriched (ASpo), polysaccharides (ASsu) or residue (ASre) fractions.

In the MTT and CCK-8 assays, only ASpo substantially reduced MCF-7 and MDA-MB-231 cell growth and proliferation, comparable to *A. seyal* hydro-ethanolic extract. MTT (Mitochondrial tetrazolium test) and CCK-8 (Cell Counting Kit-8) assays are two standard colorimetric methods commonly used to screen lead drug candidates in drug discovery through the *in vitro* measurement of cell viability, proliferation and cytotoxicity (Shekhawat et al., 2025). The CCK-8 assay, does not require dimethyl sulfoxide (DMSO) dissolution of formazan crystals from MTT dye with a reaction that produces a water-soluble formazan product, making the method faster, easier to perform, and more sensitive than MTT (Shekhawat et al., 2025; Fan et al., 2024). Thus, the significant decrease of MDA-MB-231 and MCF-7 cell growth only obtained with ASpo similarly to crude hydro-ethanolic extract, confirm our previous observations (Zingue et al., 2018a; Zingue et al., 2018b). The activity of the whole extract appears predominantly attributable to its polyphenolic constituents. These results are in agreement with the works of Vuong et al. (2016) and Hilary et al. (2021), who also reported in just proofread thoroughly and highlight the changes made both MTT and CCK-8 assays polyphenol-enriched preparation from European blueberry (200 μM gallic acid equivalent) after 24 h of exposure and seeds extract of *Phoenix dactylifera* L. (date) rich in polyphenols (250 μg/mL) after 48 h of exposure, a significant inhibition of MCF-7 and MDA-MB-231 cell proliferation. Moreover, the obtained results showed that the triple-negative MDA-MB-231 cells are more sensitive to ASpo as they were also with the crude extract (Zingue et al., 2018a).

The colony formation assay definitively determines through a direct measure (cell counting) the long-term effects of cytotoxic agents on the survival and proliferation of cancer cells or clonogenic survival and tumorigenic potential (Brix et al., 2021). In the present study, the colony formation assay showed that ASpo significantly and concentration-dependently reduced the number of clones of triple-negative MDA-MB-231 cells. This definitively confirms that ASpo contributed *in vitro* to the cytotoxicity of *A. seyal* hydro-ethanolic extract on triple-negative MDA-MB-231 cells which could be suggested to be more sensitive to this fraction. Moreover, the potential of ASpo to inhibit *in vivo* mammary tumorigenesis could be hypothesized at first glance based on its inhibitory activity against MDA-MB-231 cancer cell clones. Similar results were obtained with Korean *Artemisia annua* L. polyphenolic extract, which significantly reduced the triple-negative MDA-MB-231 cell colonies at concentrations of 10, 50 and 100 μg/ml (Ko et al., 2020). The *A. seyal* hydro-ethanolic extract at 132.5 μg/ml highlighted intrinsic apoptosis as a cell death mechanism linked to this cytotoxicity through the significant increase in caspase nine and caspase three activities. A similar result was also observed in the present study with ASpo, which increased caspase-3 activity in triple-negative MDA-MB-231 cells at the lower concentrations (50 and 100 μg/ml). Polyphenolic extract of Italian *Annurca* apple at 300 μM catechin equivalent also induced the cytotoxicity in triple negative MDA-MB-231 cancer cells through activation of the initiator for intrinsic or mitochondrial pathway of apoptosis-related cell death (caspase 9) and effect or caspase 3 with confirmation by significant increase of ratio pro-apototic Bax protein over anti-apoptotic protein Bcl-2 (Martino et al., 2019). It appears that polyphenols are responsible for the main effect of crude extract, which was previously demonstrated to trigger the intrinsic apoptosis as cell death mechanism by activating the caspase nine and caspase-3 activities, and downregulated anti-apoptotic proteins Bcl-XL and Bcl-2, although not investigated in this study (Zingue et al., 2018b).

In addition to the activation of the apoptotic effector caspase 3, ASpo induced on triple-negative MDA-MB-231 cells a significant increment of collagen and fibronectin adhesion and a reduction of cell migration and cell invasion. The crude hydro-ethanolic extract from which ASpo was derived showed after 48 h at the high concentration (265 μg/ml) a significant inhibition of MDA-MB-231 cell migration and at concentrations of 66 μg/mL, 132.5 μg/mL and 265 μg/mL a significant inhibition of invasion (Zingue et al., 2018a). Thus, polyphenols appear to be the active ingredients responsible for the anti-invasive activity of *A. seyal* hydro-ethanolic extract on MDA-MB-231 triple-negative cells, and ASpo would possess anti-migration activity at a concentration almost 5 times lower (50 μg/ml versus 265 μg/ml). This result is in line with the works of Ko et al. (2015) and Wu et al. (2017), who reported a significant anti-invasion activity with *Euphorbia supina* polyphenols (5 μg/ml) and *N. nucifera* leaf polyphenol extract (100 μg/ml), respectively.

Summing up *in vitro*, of the three *A. seyal* fractions, ASpo is the one that would contribute to *in vitro* anti-cancer activity of crude *A. seyal* hydro-ethanolic extract in our works due to its anti-proliferative, anti-clonogenic, apoptotic and anti-metastatic properties on MDA-MB-231 cells. However, this *in vitro* system useful for high-capacity screening of new anticancer substances with these isolated breast cancer cells in two-dimensional (2D) culture have limitations because it does not reproduce *in vivo* complex tissue with extra-cellular matrix interactions of solid tumor’s micro-environment in a dynamic living organism with physiological barriers (Bytautaite and Petrikaite, 2020). Furthermore, MDA-MB-231 cells with higher metastatic potential come from a triple-negative breast cancer which accounts for 15%–20% of breast cancer subtypes, while MCF-7 cells come from an estrogen-dependent breast cancer known as the most frequent subtype (65%–70%) globally with conventional tamoxifen and letrozole as preventive drugs in spite of undesirable hot flashes and endocrine resistance (Zeng et al., 2022; Benesova et al., 2024). This estrogen-dependent breast cancer experimentally induced in Wistar rats by sufficiently referenced pre-carcinogen DMBA is in addition the only well-established *in vivo* breast cancer model (Zingue et al., 2018a; Wang et al., 2021; Yadav and Chumbhale, 2022; Fotsing et al., 2023). To follow up, ASpo has been assessed in DMBA-induced breast cancer in rats.

As key results of this second study, ASpo, particularly at the higher dose (37.5 mg/kg), similarly to reference drugs, increased the survival rate of rats and markedly reduced the incidence, burden, and volume of mammary tumors, which were characterized as SBR II ductal carcinomas with 30% lymphocytic infiltration, compared to the DMBA group. The subcutaneous injection (*s.c*) of a single dose (50 mg/kg) of the pre-carcinogen DMBA, is known to directly target mammary tissue and be metabolized by cytochrome P450 enzymes into epoxides including DMBA -3,4-dihydrodiol-1,2-epoxide mutagen (Wang et al., 2021). This epoxide in association with oxidative stress and chronic inflammation, induces mammary tumors or estrogen-dependent mammary cancer (ductal carcinoma cribriform, comedo, or papillary similar to human ductal carcinoma) in peripubertal rats (45–55 days) with a high rate of proliferation and differentiation of terminal end buds into alveolar buds and terminal ducts (Russo, 2015; Yadav and Chumbhale, 2022). The severity of this tumor development (tumor burden) in rats with DMBA-induced immunosuppression has been associated with a decrease in the survival of rats (Gao et al., 2008). This suggests that ASpo may exert a protective (immunostimulatory) effect against the toxicity of the DMBA pre-carcinogen, with anti-proliferative and preventive actions on DMBA-induced estrogen-dependent mammary carcinogenesis. These anti-proliferative actions were also observed previously with ASpo associated with significant inhibition of estrogen dependent MCF-7 cell growth (MTT assay) and reduction of cell proliferation (CCK-8 assay). The hypothesis stated with interpretation of clonogenic findings about the potential of ASpo to inhibit *in vivo* mammary tumorigenesis appears to be confirmed. These results are also in line with the works of Kumaraguruparan et al. (2007) and Liu et al. (2009), who likewise reported preventive effects on DMBA-induced mammary tumor development in *Sprague-Dawley* rats with supplementation of Indian black tea polyphenols and United States of America apple extract rich in polyphenols. Thus, ASpo appears to contribute to the anticancer activity of the crude hydro-ethanolic extract, which, at both doses (150 and 300 mg/kg), reduced the incidence and volume of mammary tumors at least four times more than the fraction (37.5 mg/kg) (Zingue et al., 2018a). The anti-proliferative activities of ASpo against estrogen-dependent breast cancer cells, specifically the MCF-7 line, has been demonstrated above and suggest the involvement of anti-estrogenic activities that should also be investigated. However, ASpo has been investigated on biomarkers of oxidative stress and inflammation also involved in DMBA-induced mammary tumorigenesis (Wang et al., 2021).

Oxidative stress resulting from damage to endogenous antioxidant defenses (SOD, CAT, GSH, glutathione peroxidase) promotes DMBA-induced mammary carcinogenesis with DNA mutations by DMBA epoxides, reactive oxygen species (O_2_•−, HO•, and H_2_O_2_) and nitrogen oxide species (NO_2_, ONOO−) with nitric oxide (NO) following El Makawy et al. (2022). Thus, ASpo that significantly increased SOD and catalase activities and reduced nitric oxide (NO) and MDA levels in dose dependent fashion would possess antioxidant potential, which could contribute to explaining its 50% reduction in tumor incidence. These results are in agreement with those of Kumaraguruparan et al. (2007) and Cuevas-Rodríguez et al. (2010), who respectively observed an increase in SOD and CAT activities with polyphenols from Indian black tea in *Sprague-Dawley* rats exposed to DMBA, and a decrease in NO level with Mexican blackberries rich in polyphenolics.

DMBA which induces oxidative damage of the mammary gland with epoxides is known to activate the transcription factor NF-κB (nuclear factor-kappa-B) that modulates genes’ expression involved in inflammation including cytokines (Liu et al., 2017). The balance between pro-inflammatory cytokines (TNFα, IL-1β, IL-2, IL-6, IL-8, IL-12, and IFN-γ) and anti-inflammatory cytokines (IL-1RA, IL-4, IL-10, IL-13, and TGFβ) is considered to be a key factor in immune response homeostasis and inflammatory process associated with numerous diseases (Bohstam et al., 2017). IFN-γ is an effector cytokine central to cancer immunosurveillance, exerting pleiotropic effects through the canonical JAK/STAT signaling pathway and promoting tumor cells apoptosis by upregulating the expression of caspase 3, 8 (Castro et al., 2018). Then, ASpo that remarkably decreased serum levels of TNF-α, IL-6, IL-12 and EGF and increased IFN-γ especially at higher dose (37.5 mg/kg) could be suggested to exert strong anti-inflammatory effects in DMBA-induced mammary tumorigenesis, potentially inhibiting tumor development through the immunomodulatory effects with tumoricidal or apoptotic actions of IFN-γ. These results are supported by González et al. (2011) who reported that polyphenols reduce macrophages activity by downregulating cyclooxygenase-2 (COX-2), inducible nitric oxide synthase and decrease then expression of TNF-α, IL-1-β and IL-6.

The results observed on pro-inflammatory cytokines with ASpo of this study are in phase with the increase in blood monocytes (15.42% vs. 6.32% in DMBA) and the decrease in blood granulocytes (16.3% vs. 27.62% in DMBA). These results are also in phase with the lymphocytic infiltration of the breast tumor (30% vs. 10% in the DMBA group) aligned with the high blood lymphocyte count (74% vs. 66% in the DMBA group) supported by the increased relative mass of the thymus and its cortical density in the histoarchitecture. These statements are supported by Eren et al. (2024) that highlighted the immunomodulatory effects of polyphenols in prevention and treatment of breast cancer.

The previous anti-breast cancer mechanisms of ASpo are supporting by polyphenolic compounds detected, namely, caffeic acid known for its free radical scavenging and antioxidant activities (Khan et al., 2016). da Cunha et al. (2004) demonstrated its ability to inhibit the increase of pro-inflammatory cytokine such as IL-1β and iNOS enzyme and to exhibit anti-inflammatory and immunomodulatory activities with caffeoylquinic acids (Chang and Lin, 2025). Gallic acid also detected inhibited breast cancer cell growth (Rezaei-Seresht et al., 2019), while caffeic acid reduced mouse breast tumor through immunomodulation via M1 macrophage (Xie et al., 2024).

In addition to understanding the mechanisms of action, it was also essential to investigate the potential toxicological or adverse effects of ASpo following 20 weeks of treatment. As results, no significant variation in the weight of the animals during the experiment was observed with ASpo. On the hematological parameters, the variation in monocytes, lymphocytes, and granulocytes would be linked to the immunomodulatory activities of the fraction. Compared to the DMBA group, the increase in red blood cells close to the value of the normal group and the hematocrit or percentage for the volume of red blood cells in whole blood observed with this low-dose of ASpo (18.75 mg/kg) would be linked to its antioxidant properties (Behl et al., 2020). These properties would protect red blood cells against hemolysis induced by the oxidative stress of DMBA (Shivani et al., 2025). The mass of an organ in toxicity studies is an important indicator of the deleterious effects of chemical substances or an experimental compounds (Lazic et al., 2020). Upon analyzing certain organs in the study (relative mass and histoarchitecture), the variations observed in the thymus, femur and spleen were correlated with the immunomodulation of ASpo and DMBA-induced inflammation. The decrease by ASpo (18.75 mg/kg) of the relative mass of uterus, an estrogen-sensitive organ, could be linked to its anti-estrogenic activities hypothesized with the inhibition of the growth of estrogen-sensitive MCF-7 cells. The dose-dependent restoration towards normal compared to DMBA group of adrenal glands and heart’s relative mass with ASpo at the tested doses (18.75 and 37.5 mg/kg) could be attributed to these antioxidant and anti-inflammatory protective effects on the heart and the known adrenocorticolytic effects of DMBA (Li and Robaire, 2025). These protective effects are supported by Stankovic et al. (2025) that referenced plant polyphenols through cardioprotective effects as best friends for the heart. The increase in the relative mass of the ovaries observed with the high dose of ASpo (37.5 mg/kg) compared to DMBA group would be related to the increase in the number of ovarian follicles as reported by Maranesi et al. (2021) with olive polyphenols on rabbit ovaries. An increased serum creatinine can be an indicator of kidney dysfunction while a substantially increased serum alanine transaminase (ALT) is considered as biomarker of liver toxicity (Liu et al., 2014; Ávila et al., 2025). The preserved histo-architecture of the liver and kidneys observed with ASpo, along with non-significant differences in ALT activity and creatinine level, suggests that this fraction is non-toxic with protective effects. These results are supported by previous observation with the crude extract (Zingue et al., 2018b).

To finish with the lacking items of this study, it would have been interesting to evaluate the effects of ASpo on caspase 9, anti-apoptotic proteins such as Bcl-XL and Bcl-2 to confirm that it acts through intrinsic apoptosis observed with *A. seyal* crude extract on previous studies (Zingue et al., 2018a; Zingue et al., 2018b). Moreover, although *in vitro* studies have shown that ASpo is more active on MDA-MB-231 cells than on MCF-7 cells, the cancer model used *in vivo* is one that replicates estrogen-dependent cancers type. The use of an *in vivo* triple negative xenograft breast cancer model would bring more light on the anticancer potential of ASpo. *In silico* study could be further used to speculate on the ADME profile of ASpo.

## 5 Conclusion

Overall, polyphenol-enriched fraction of *A. seyal* (ASpo) compared to the polysaccharide (ASsu) and residue (ASre) fractions is the one that retained total extract activity and contributed to its *in vitro* and *in vivo* anti-breast cancer activities with safety profile. This fraction alone inhibited cell growth, cell proliferation and clone formation through apoptosis, increase of collagen and fibronectin cell adhesion and inhibition of cell invasion and migration. *In vivo* on estrogen-dependent breast cancer induced by DMBA in Wista*r* rats, ASpo inhibited incidence, burden and mammary tumor volume particularly at the high dose (37.5 mg/kg). These preventive effects would involve antioxidant, anti-inflammatory and immunomodulatory effects. Isolation of specific polyphenolic compounds including quantified flavonoids and flavonols responsible for observed effects is also an important gap for future research on Cameroonian *A. seyal*.

## Data Availability

The raw data supporting the conclusions of this article will be made available by the authors, without undue reservation.
